# Auditory motion perception emerges from successive sound localizations integrated over time

**DOI:** 10.1038/s41598-019-52742-0

**Published:** 2019-11-11

**Authors:** Vincent Roggerone, Jonathan Vacher, Cynthia Tarlao, Catherine Guastavino

**Affiliations:** 1 Centre for Interdisciplinary Research in Music Media and Technology, Multimodal Interaction Laboratory, McGill University, Montreal, Canada; 2 Department of Systems and Computational Biology, Albert Einstein College of Medicine, New-York, USA

**Keywords:** Perception, Biophysical models, Emergence, Signal processing

## Abstract

Humans rely on auditory information to estimate the path of moving sound sources. But unlike in vision, the existence of motion-sensitive mechanisms in audition is still open to debate. Psychophysical studies indicate that auditory motion perception emerges from successive localization, but existing models fail to predict experimental results. However, these models do not account for any temporal integration. We propose a new model tracking motion using successive localization snapshots but integrated over time. This model is derived from psychophysical experiments on the upper limit for circular auditory motion perception (UL), defined as the speed above which humans no longer identify the direction of sounds spinning around them. Our model predicts ULs measured with different stimuli using solely static localization cues. The temporal integration blurs these localization cues rendering them unreliable at high speeds, which results in the UL. Our findings indicate that auditory motion perception does not require motion-sensitive mechanisms.

## Introduction

One of the major challenges to the auditory system is to track moving sound sources to predict their path and guide action (*e.g*. avoid an approaching car). Yet, our understanding of auditory motion mechanisms is lagging compared to our understanding of static sound localization. Motion-induced changes in acoustical cues include frequency shifts (Doppler effect^1^), variations of intensity (Looming effect^2^), and motion parallax^3^. However, the existence of motion-sensitive mechanisms in the auditory system, similar to motion detectors found in the visual system, is still an open debate (see Carlile & Leung^4^ for a comprehensive review).

There is psychophysical evidence that distance and duration are the primary cues for auditory motion perception^5^. While speed can be used when distance and duration cues are unreliable, it does not dominate in audition as it does in vision^5^. At a neurophysiological level, different cortical responses have been observed for static and moving sounds^6,7^, but it remains unclear whether these responses reflect explicit motion sensitivity or rather sensitivity to changes in spatial position^4^. Along those lines, Grantham proposed the early snapshot theory (now referred to as 2-point snapshot model)^8^ positing that speed is estimated from the comparison of the successive positions of the sound at the starting and end points. Perrott extended this view with a multi-snapshot model to account for speed variations during motion^9^. However, this model does not account for the sensitivity to fine-grain speed variations without considering temporal integration mechanisms^10^.

One effect thought to be a direct consequence of a minimal integration time is the existence of Upper Limits for circular auditory motion (UL)^11,12^. The UL is defined as the speed (in rot/s) above which listeners fail to identify the direction of sounds spinning around them. The study of circular trajectories provides a unique paradigm to resolve alternative explanations for motion perception as it involves changes in azimuthal position while excluding motion-induced acoustical cues.

The present study investigates the perceptual mechanisms at play to track moving sounds. To do so, we measure the upper limit for auditory motion perception for stimuli with different spectral content. Our data reveal that the UL increases with the center frequency and bandwidth of the stimulus (Experiments 1 and 2), and that the UL originates from front-back confusions (Experiment 3). These empirical results lead us to propose a model that accounts for variations in UL as a function of the spectral content of the stimulus. The proposed model is based on static localization models^13,14^: positions are continuously tracked by the auditory system. Yet, as previously hypothesized, a minimum integration time (MIT) is required to achieve optimal performance during moving sound localization (around 300 ms^8,15^). As a consequence, localization cues are blurred by the motion of moving sounds. Such a blur can be compared to the motion blur documented in vision and computer vision^16,17^. To the best of our knowledge, our model of auditory motion is the first to reconcile previous physiological and psychological evidence using a spatial snapshot model with temporal integration.

## Results

### Experimental results

We report three experiments estimating the upper limit (UL) for circular auditory motion perception as a function of the spectral content using filtered noises. The UL is defined as the speed (in rot/s) above which participants fail to identify the direction of sounds spinning around them. It is estimated using an adaptive two-alternative-forced-choice paradigm, where the participants have to indicate the direction in which the sound is moving.

#### Experiment 1

**Figure 1:**
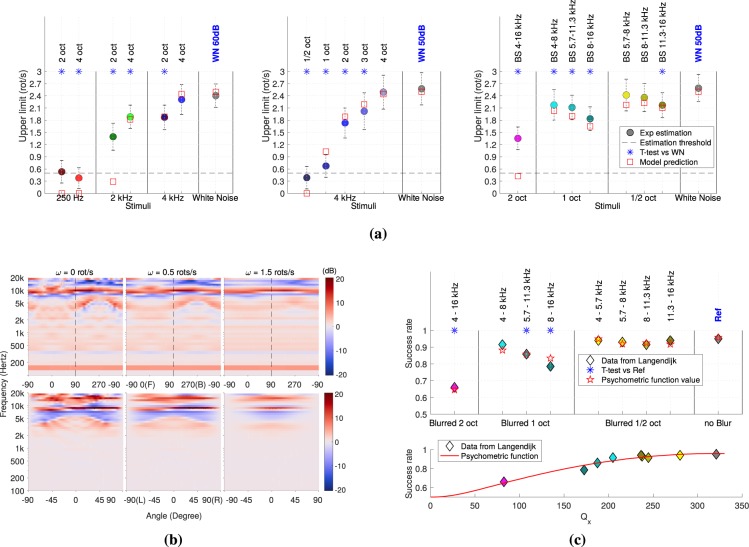
All simulations were performed using the HRIR measurements of TK audiogroup using the Diffuse Field Common Method (http://audiogroup.web.th-koeln.de/ku100hrir.html^25^). (**a**) Results of Experiments 1, 2 and 3 and associated prediction of the model, with respective Pearson correlation ${R}_{1}=0.95$, ${R}_{2}=0.97$ & ${R}_{3}=0.97$. Significant T-tests with Bonferroni correction between the reference stimulus (WN) and other stimulis are represented with blue stars. (**b**) Percept (Eq. 4) and its associated front-back difference for 3 different speeds. (F) and (B) stand for front and back directions, and (L) and (R) for left and right directions. (**c**) Front-back discrimination success rate adapted from Langendijk^13^ (Fig. 6). Rates are estimated in a headphone experiment, by smoothing HRTF frequency bands over angle, with the same cut-off frequencies as in Experiment 3 (using the same color coding as in (**a**).

In Experiment 1, we test the effect of spectral content on the upper limit by manipulating the Center Frequency (CF) and Bandwidth (BW) of band-pass filtered noises, using manipulations similar to those used by Yost & Zhong for static localization^18^. The reference stimulus is a White Noise (WN) presented at 60 dB. Six stimuli were generated using a 3 (CFs) × 2 (BWs) factorial design with three CFs: 250 Hz, 2 kHz and 4 kHz) and two BWs: 2 oct and 4 oct). The bandwidths were determined based on a pilot experiment and are wider than those used by Yost & Zhong^18^. Further details are presented in Methods section and Table 1, and discussed in the section Discussion. Results are summarized in Fig. 1a (left panel).

**Table 1:** Table of parameters of the stimuli used and the measured level of presentation for the 3 Experiments.

Label	Low cut-off freq. (kHz)	High cut-off freq. (kHz)	Sound Pres. Level (dBA)	Initial speed (rot/s)
**Exp 1: Band-pass filter**				
250 Hz 2oct	0.1	0.6	61	0.5
250 Hz 4oct	0.06	1.06	65	0.5
2 kHz 2oct	0.83	4.83	65	0.9
2 kHz 4oct	0.47	8.47	63	0.9
4 kHz 2oct	1.65	9.66	62	1.3
4 kHz 4oct	0.94	16.94	58	1.3
**Exp 2: Band-pass filter**				
4 kHz 1/2oct	3.1	5.1	55	0.5
4 kHz 1oct	2.5	6.5	51.5	0.5
4 kHz 2oct	1.6	9.6	50.3	1.3
4 kHz 3oct	1.2	13.2	49.5	1.3
4 kHz 4oct	0.9	16.9	48.2	1.3
**Exp 3: Band-stop filter**				
BS 4–16 kHz	4	16	50.1	0.9
BS 4–8 kHz	4	8	49.9	1.3
BS 5.7–11.3 kHz	5.7	11.3	50.4	1.3
BS 8–16 kHz	8	16	51.0	1.3
BS 5.7–8 kHz	5.7	8	49.8	1.3
BS 8–11.3 kHz	8	11.3	49.7	1.3
BS 11.3–16 kHz	11.3	16	50.5	1.3

Levels were adjusted to have the same perceived level.

A 3 (CF) × 2 (BW) factorial repeated-measure ANOVA over all participants revealed significant main effects of CF (*F*(2, 20) = 537.9, *p* < 10^−4^) and BW (*F*(1, 10) = 20.7, *p* < 10^−3^). There was also a significant interaction effect between CF and BW (*F*(2, 20) = 10.1, *p* < 10^−3^). However, the interaction effect can be attributed to the low CF stimuli (250 Hz), which yielded invalid estimations below the 0.5 rot/s threshold described in Materials and Method.

*Posthoc* For the main effects, T-tests with Bonferroni correction revealed significant differences between the reference stimulus (WN) and all other stimuli except for ‘4 kHz 4 oct’. Results indicate that the UL increases with BW and CF. However, stimuli with larger BWs also have more high frequency content, which could be a confounding factor. Experiment 2 was designed to further investigate the effect of bandwidth by testing a wider range of bandwidths centered at 4 kHz.

#### Experiment 2

In experiment 2, we clarify the effect of BW by testing a wider range of BWs for the particular CF of 4 kHz for which performance was higher in Experiment 1. Five stimuli were generated with BWs: 0.5 oct, 1 oct, 2 oct, 3 oct and 4 oct. The reference stimulus is now a WN presented at 50 dB to keep a comfortable level. Further details are presented in Materials and Method and Table 1, and discussed in section Discussion. Results are summarized in Fig. 1a (center panel).

A repeated-measure ANOVA over all participants with Greenhouse-Geisser correction revealed a significant main effect of BW ($F(2.5,35.7)=89.4$, $p < {10}^{-4}$).

*Posthoc* T-tests with Bonferroni correction revealed significant differences between the reference stimulus (WN) and all other stimuli except for ‘4 kHz 4oct’. Performance for the stimuli ‘4 kHz 1/2oct’ and ‘4 kHz 1oct’ is around the 0.5 rot threshold described in Materials and Method. In comparison, in Exp. 1, performance for the stimulus for ‘2 kHz 2oct’ containing the same high frequency content was around 1.4 rot/s. Results confirm that the UL increases with BW, and exclude the confounding effect of high frequency content.

Post-questionnaires with participants of Experiment 1 and 2 lead us to hypothesize that the UL mainly comes from front-back confusions, as discussed in subsection Model of section Results. To test this hypothesis, we designed Experiment 3 based on previous research on front-back confusions in static sound localization^13^.

#### Experiment 3

In Experiment 3, we test the link between the UL and front-back confusions rates using band-stop filtered noises. The reference stimulus is a WN presented at 50 dB. Seven stimuli were generated using a logarithm spacing of the cut-off frequencies, to cover the target range of [4 kHz, 16 kHz] as a function of the BW considered, based on the manipulations previously used to investigate front-back confusions^13^ (Filters are shown in the Supporting Information Fig. S2a). Since we presented stimuli over a loudspeaker array, we did not reproduce Langendijk^13^ manipulations exactly. Indeed, we removed the frequency band whereas Langendijk averaged the frequency band in the simulated Head Related Transfer Function (HRTF) using binaural headphone presentation. Further details are presented in the Methods section and Table 1, and discussed in the Discussion section . Results of Langendijk are presented in the Fig. 1c. Results of Experiment 3 are summarized in Fig. 1a (right panel).

A repeated-measure ANOVA over all participants with a Greenhouse-Geisser correction revealed a significant main effect of BW on the UL ($F(3.2,54.2)=32.3$, $p < {10}^{-4}$).

*Posthoc* T-tests with Bonferroni correction revealed significant differences between the reference stimulus (WN) and all other stimuli except for ‘BS 5.7–8 kHz’ and ‘BS 8–11.3 kHz’. Results indicate that the UL decreased as the CF and the BW of the band-stop filter increased. The performance closely matches the front-back confusion rates reported by Langendijk^13^, as shown in Fig. 1c, providing support for our hypothesis.

### Model

#### Front-back discrimination for sound in motion

A majority of subjects (Exp 1: 6/11, Exp 2: 6/16, Exp 3: 14/18) reported localizing the sound at the very beginning and very end of each trial and trying to determine if the sound passed by in front or behind them. This is consistent with the observation made by Aschoff^19^: at speeds above the UL, participants no longer perceive trajectories but only the left-right alternations. In addition, many participants (Exp 1: 9/11, Exp 2: 10/16, Exp 3: 11/18) also reported modified trajectories (half-circle or eight figure) and/or ‘jumps’ between positions, characteristic of front-back confusions. This relationship between front-back confusion rates and the UL is confirmed by the results of Experiment 3. Based on these findings, we hypothesize that the UL is mainly governed by front-back discrimination performances.

The interaural cues (Interaural Time and Level Differences, resp. ITD and ILD) used for static localization do not allow to discriminate between front and back because they are approximately symmetrical with respect to the midline formed by the two ears^20^. Instead, front-back discrimination relies on spectral patterns resulting from diffraction by the torso, head and pinna. These spectral patterns are incorporated into our HRTFs and differences in HRTFs between sound sources in the front and in the back have been shown to be the dominant cue to resolve front-back confusions in static localization tasks^13,14^. However, the results of Experiment 3 suggest that front-back confusions rates are affected by speed. We posit that the estimation of front-back differences for moving sounds is blurred due to existence of a minimal integration time (MIT), rendering this cue unreliable at high speeds. This view is consistent with the sluggishness of the binaural system^15^ and reflects the same temporal integration mechanisms as motion blur in vision.

In the following section, we detail the proposed model. To the best of our knowledge, this is the first model accounting for temporal integration in auditory motion perception.

#### Mathematical formulation

*Internal representation of sound.* A sound *x* is first filtered by the HRTF filter corresponding to the angular position *θ* of its source. Then it is filtered by the gammatone filter bank that models cochlear filtering^21^. Therefore, we assume that the sound *x* is internally represented by a collection of energy levels ${({E}_{r}^{x}(\theta ,n),{E}_{l}^{x}(\theta ,n))}_{n\in \{1,\ldots ,N\}}$ where *n* denotes the frequency band (corresponding to a gammatone filter) and $\{r,l\}$ denotes the right and left ears. One has

1 $$\forall u\in \{r,l\},{E}_{u}^{x}(\theta ,n)=\mathop{\int }\limits_{f\in  {\mathcal F} }|{\hat{i}}_{u}(\theta ,f)\hat{x}(f){\gamma }_{n}(f){|}^{2}{\rm{d}}f$$

where ${\mathcal F} =[20,20000]$ is the frequency domain, ${\hat{i}}_{u}$ and $\hat{x}$ are respectively the Fourier transform of the right (or left) ear HRIR *i*_*u*_ (*i.e*. the HRTF) and the Fourier transform of sound *x* and *γ*_*n*_ is the *n*^th^ gammatone filter^22,23^

2 $${\gamma }_{n}(f)=\frac{1}{{(1+j\frac{f-{f}_{n}}{{b}_{n}})}^{4}}\,{\rm{w}}{\rm{i}}{\rm{t}}{\rm{h}}\,\{\begin{array}{c}{f}_{n}={f}_{n-1}+{b}_{n-1}\\ {b}_{n}=0.108{f}_{n}+24.7\end{array}$$

with (*f*_*n*_, *b*_*n*_) being the gammatone CF and BW. To cover the complete frequency range, we use $N=43$ filters between CFs ${f}_{1}=50\,{\rm{Hz}}$ and ${f}_{N}=19.4\,{\rm{kHz}}$. This logarithmic sampling accounts for the Weber’s law of frequency perception. In order to also account for the Weber’s law of sound amplitude perception, we consider the log-energy level of the two ears

3 $${e}_{x}(\theta ,n)=20\,{\log }_{10}({E}_{r}^{x}(\theta ,n)+{E}_{l}^{x}(\theta ,n)).$$

If the sound direction is far away from the midline we use a single ear to discriminate front and back^13^. In this case, the energy received by the contralateral ear tends toward zero compared to the power received by the ipsilateral ear. The sum in Eq. 3 reflects this behavior, binaural weighting is therefore not necessary in our model (as in^14^). Distinction between front and back is supposed to be robust to the average sound amplitude, to this purpose we compute the gradient of the log-energy^14^

4 $${P}_{x}(\theta ,n)={e}_{x}(\theta ,n)-{e}_{x}(\theta ,n-1).$$

These energy gradients ${P}_{x}(n,\theta )$ are represented in Fig. 1b –top-left (*ω* = 0 rot/s). Frequencies are interpolated for a proper gradient computation (see Supporting Information).

*The front-back cue.* In order to characterize the front-back discrimination, we summarize the front-back internal representation as a real number

5 $${Q}_{x}={\int }_{\theta \in {I}_{f/b}}\sum _{n\in {\mathscr{N}}}|{P}_{x}(\theta ,n)-{P}_{x}(180-\theta ,n)|{\rm{d}}\theta $$

where $\theta \in {I}_{f/b}=[\,-\,90,90]$ and ${\mathscr{N}}=\{2,\ldots ,N\}$. The front-back differences $|{P}_{x}(\theta ,n)-{P}_{x}(180-\theta ,n)|$ are represented in Fig. 1b –bottom-left (*ω* = 0 rot/s). Intuitively, the variable *Q*_*x*_ summarizes the saliency of the front-back discrimination cues for a sound *x*. This scalar variable is connected to the front-back confusion rate through a psychometric function. This is exemplified in Fig. 1c on the front-back confusion rates measured by Langendijk *et al*.^13^ (see Supporting Information). Therefore, we consider *Q*_*x*_ as the main observer’s internal cue to resolve front-back confusion.

*Effect of speed on the front-back cue.* The existence of a MIT causes the auditory system to accumulate information over a duration *T*_*i*_. When the sound *x* is revolving around a participant with a speed *ω* (in rot/s), the duration *T*_*i*_ corresponds to a traveled angle ${\Delta }_{\omega }=2\pi \omega {T}_{i}$. It follows that the sound *x* is internally represented by a collection of energy levels ${({E}_{u}^{x}(\theta ,n))}_{\theta }$ (Eq. 1) coming from the angular sector Δ_*ω*_ centered at *θ*, instead of a single direction *θ*. These energy levels can be summarized by their average value *i.e*. $\forall \,u\in \{r,l\},$

6 $${E}_{u}^{x}(\omega ,\theta ,n)=\frac{1}{{\Delta }_{\omega }}{\int }_{0}^{2\pi }{E}_{u}^{x}(a,n){1}_{{\Delta }_{\omega }}(a-\theta ){\rm{d}}a$$

where ${1}_{{\Delta }_{\omega }}$ is the indicator function of $[0,{\Delta }_{\omega }]$. The energy levels defined in Eq. 6 are blurred versions of the energy level defined in Eq. 1. From these energy levels ${E}_{u}^{x}(\omega ,\theta ,n)$, we define the log-energy level ${e}_{x}(\omega ,\theta ,n)$, the gradient of the log-energy ${P}_{x}(\omega ,\theta ,n)$ and the front-back internal representation *Q*_*x*_(*ω*) following Eqs 3, 4 and 5 respectively. The energy gradients ${P}_{x}(\omega ,\theta ,n)$ and their front-back differences $|{P}_{x}(\omega ,\theta ,n)-{P}_{x}(\omega ,180-\theta ,n)|$ are represented in Fig. 1b –middle and right ($\omega =0.5\,{\rm{and}}\,1.5\,{\rm{rot}}/{\rm{s}}$). As the blurring reduces the differences between front and back, the function $\omega \mapsto {Q}_{x}(\omega )$ monotonically decreases. It reaches 0 for *ω* = 1/*T*_*i*_, which is the point where the width of the rectangular function is equal to 2*π* (see Supporting Information Fig. S2b).

We hypothesize that to resolve front-back confusions, the value *Q*_*x*_(*ω*) needs to reach a threshold *Q*_min_. The value of *Q*_min_ can be evaluated experimentally using the UL measured under the reference condition *i.e*. a WN rotating at speed ${\omega }_{{\rm{UL}}}^{{\rm{WN}}}=2.5\,{\rm{rot}}/{\rm{s}}$^12,24^, therefore ${Q}_{{\rm{\min }}}={Q}_{{\rm{WN}}}({\omega }_{{\rm{UL}}}^{{\rm{WN}}})$. Finally, we define the predicted UL ${\omega }_{UL}$ for a test sound *x* by

7 $${\omega }_{UL}={Q}_{x}^{-1}({Q}_{{\rm{\min }}}).$$

## Discussion

### Perception of trajectories and localization

Yost & Zhong^18^ reported that localization accuracy increased with BW for any CF. For the highest BW (2 oct) they tested, they reported ceiling performance for all CFs. The authors discussed these findings in terms of availabilty of both ITD and ILD cues. In contrast, we observe that the UL decreases with the BW (Fig. 1a –middle) for intermediate and high CFs (2 kHz and 4 kHz, Fig. 1a –left). We solve this apparent contradiction by showing that the UL is governed by front-back discrimination rather than by ILD and ITD cues. This indicates that the recognition of spectral patterns is an additional necessary step to identify sound motion direction.

Our model fails to predict two UL values (Fig. 1a conditions 2 kHz–2 oct and BS 4–16 kHz). These values are obtained under similar conditions: both stimuli have a high cut-off frequency around 4.5 ± 0.5 kHz and a low cut-off frequency under 1 kHz, where spectral patterns are almost uninformative (Fig. 1a,b for a CF of 250 Hz). As a result, both stimuli have a weak front-back saliency (low *Q*_*x*_). Since our model only takes into account spectral patterns, UL predictions are close to 0 for both conditions. We propose two explanations for these discrepancies. First, our model does not consider ILD cues. Yet, because of their asymmetry with respect to the midline (ears have a frontal orientation), they could help resolve front-back confusions and therefore identify sound motion direction. Second and to a lesser extent, the HRTFs used in our model^25^ are obtained with a dummy head – which does not account for shoulder and torso effects or individual differences - both of which could provide additional cues in low frequencies.

The very low UL values obtained under the lowest frequency conditions (Fig. 1a, CF of 250 Hz) where only ITD cues are available confirms that ITD cannot help to resolve front-back confusions.

### Spectral pattern recognition sluggishness

**Figure 2:**
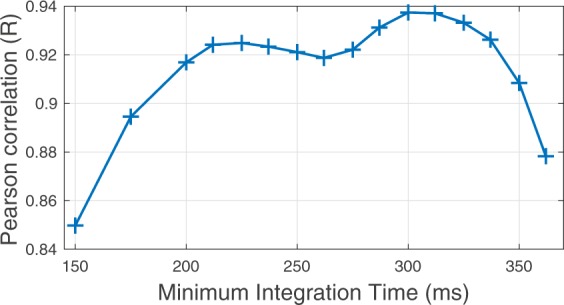
Pearson correlation coefficient ($0\le R\le 1$) between model and data for all experiments as a function of MIT.

Importantly, we combine the MIT with front-back confusions to explain the UL. Maximal correlation between predictions and data is obtained with previously proposed MIT *e.g*. 300 ms^8,15^ (see Fig. 2) demonstrating the relevance of this combination.

The MIT was previously related to the sluggishness of binaural system^15^. Because of the energy summation of the two ears in Eq. 3 our model is binaural. We make this choice for computational simplicity (see Supporting Information). However, spectral pattern recognition can be performed independently and in parallel for the two ears. Despite not being binaural, the estimation of a reliable spectral pattern still requires time. Therefore, we propose that this sluggishness is primarily due to the integration time required to achieve reliable estimates rather than to the combination of binaural cues^26^.

### A temporally-integrated snapshot model

By combining the MIT with front-back confusions, we bridge the gap between static localization and motion perception. Such a connection favors a multi-snapshot model where the sound location is sampled over space (not time) with a MIT.

This model is in accordance with Locke *et al*.’s results showing that in their experiment “listeners were highly insensitive to instantaneous increases in velocity”^10^. Such an insensitivity is due to the high value of the MIT (300 ms) compared to the duration of their stimuli which prevents the listeners to evaluate continuous speed changes.

In addition, our model is helpful to understand the relation between the Mininum Audible Angle (MAA) and the Minimum Audible Movement Angle (MAMA). The MAA is the minimal angle required to distinguish two static sound sources. In a way, it represents the sampling interval of sound source locations. The MAMA is the minimum displacement angle needed to detect sound source motion. In our model, the MIT causes motion to blur localization and therefore it impairs localization accuracy. As a consequence, it is more difficult to detect a continuous change in position than to discriminate to two static positions, especially when the speed is high. Therefore our model is compatible with the view that the MAA is a lower bound of the MAMA and that the latter increases with speed^4^.

Note that our model supposes that energy levels are averaged around the actual position of the sound. For sound tracking this seems unrealistic as the brain only receives input from the previous locations. Yet, this is not a problem for our model since we later consider a front-back confusion cue by integrating over the half-circle which makes the delay negligible. However, this should be addressed further to develop a more general sound motion tracking model.

Regarding a possible neural implementation, the proposed model follows from well-established knowledge about cochlear filtering^21^. The dorsal cochlear nucleus is hypothesized to play a role in HRTFs identification. In particular, some neurons in the Dorsal Cochlear Nucleus (DCN) are sensitive to sharp spectral changes^27^. We further propose the existence of a cue *Q*_*x*_ that accounts for front-back saliency. Such cue might be directly encoded by some neurons in the DCN (or higher in the auditory hierarchy). However, this cue could also be described differently from our proposition (5) *e.g*. by the difference between independent front and back saliency cues.

### Snapshot model *vs*. motion-sensitive model

We do not present results for the alternate – motion-sensitive – model. The reasons are twofold. First, to the best of our knowledge, there is no detailed mathematical formulation in the existing literature. Second, such a model requires hypotheses that are not supported by electrophysiological literature: there is no evidence for the existence of neurons sensitive to motion only that are additionally frequency-tuned^4^.

The existence of auditory motion-sensitive channels is proposed after the observation of an Auditory Motion After Effect (AMAE)^4^. Yet, motion-sensitive channels are not necessary for the existence of an AMAE. Indeed, an AMAE could originate from adaptation mechanisms in moving sound localization which aim at predicting the future position of the sound source as previously proposed in vision^28^.

In comparison, the proposed model is based on psychophysical evidence from experiments in sound localization^13^ and auditory motion perception^15^. No further hypotheses are required. Moreover, the model predictions are obtained by only adjusting the value of *Q*_min_ such that it corresponds to the UL measured under the reference condition (WN).

### Toward a unified theory of auditory motion perception

Our data demonstrate that the direction of a moving sound can be estimated from successive, temporally-integrated snapshots. The temporal integration results in a motion blur rendering static localization cues unreliable above a certain speed. Our model accounts for the observed effect of spectral content on the upper limits. In addition, we observe a maximal correlation between model predictions and experimental results with a binaural integration time of about 300 ms, in line with previous research. We conclude that motion-sensitive mechanisms are not necessary to determine moving sound direction.

Future research includes developing a model to track sounds in space for others trajectories. We propose to move toward probabilistic models, similar to the ones developed in vision^29–31^. The probabilistic framework will allow the combination of multiple cues and prior knowledge contributing to auditory motion perception^32,33^.

## Methods

### Procedure

The three experiments estimate the upper limit (UL) for circular auditory motion perception as a function of spectral content using filtered noises. The UL is defined as the speed (in rot/s) above which participants fail to identify the direction of sounds spinning around them.

**Figure 3:**
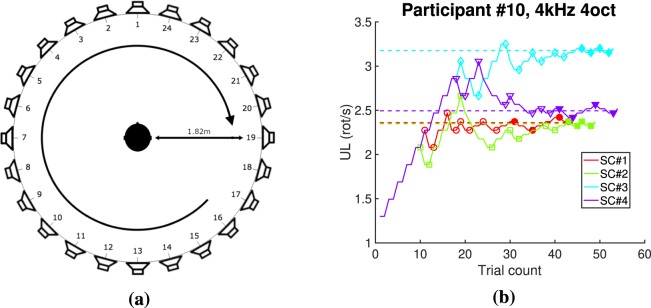
Experimental set up and staircase example. (**a**) Loudspeaker set up. (**b**) Typical staircase obtained.

On each trial, participants were asked to indicate the direction of a filtered noise spinning around them in a clockwise (CW) or counterclockwise (CCW) direction. The speed of the moving sound increases with correct answers and decreases with wrong answers. We randomized the starting point, direction and duration (see subsection Stimuli in Method section) across trials. We used an adaptive 2-up 1-down 2-alternative forced choice such that performance converged on 70.7% correct answers^34^. For each stimulus, we used 4 intertwined ascending staircases with an initial speed of 0.4, 0.9 or 1.3 rot/s (depending of the condition, based on a pilot) and an initial step size of 15% of the initial speed that was halved after the third and fifth reversals. We stopped after 12 reversals or 60 trials (whichever came first) and averaged over the last 4 reversals of the 4 staircases to estimate the UL. A typical result from one participant for one condition is shown in Fig. 3b. Each experiment consisted of several blocks of approximately 10 minutes each for each of the stimuli, including the reference stimulus (WN). We counterbalanced the order of presentation of conditions across participants.

### Stimuli

The generated sound is led by the software *MaxMSP* (Cycling ‘74’, San Francisco). Reference stimuli were created using the WN bloc in MAX/MSP at a sampling frequency of 44.1 kHz (from the bloc ‘noise’). The BLN were obtained by filtering a WN with an eighth-order Butterworth filter using the blocks ‘filterdesign’ and ‘cascade’, as in^18^. Filters are set to ‘bandpass’ for the experiments 1 and 2 and ‘bandstop’ for the experiment 3. The Butterworth filter was used as it provides a flat band, with a strong decrease outside the cut-off frequency. Levels were adjusted to have the same perceptive level. See Table 1 for parameters details.

Stimulus duration was the minimum between two random values: the times needed for the sound to do 2, 2.5 or 3 rotations and a random number between 2 s and 3 s. That way, the maximum stimulus duration is 3 s, but is much smaller for high speed trials. Note that if the speed is lower than 0.5 rot/s, the source may not do a full rotation (if the duration is close to 2 s), and therefore the UL estimation is not accurate below this threshold.

### Participants

In Experiment 1, we tested 11 participants (6 males, 5 females, Av. age 26 ± 2.7 (1 s.d)), in Experiment 2, 16 participants (12 males, 4 females, Av. age 31 ± 11.6 (1 s.d)), in Experiment 3, 18 participants (11 males, 6 females, Av. age 29.6 ± 6.7 (1 s.d.)). They received $20 CAD for their participation. Participants were instructed not to move their head during stimulus presentation. They were also informed that stimulus duration and starting position were randomized so that they could not base their answers on distance traveled. The experiments were conducted under the supervision of Prof. Guastavino from the School of Information Studies and CIRMMT and approved by the McGill Review Ethics Board (REB). All methods were performed in accordance with the Tri-agency framework, Responsible Conduct of Research (RCR, 2016)^35^. All participants gave their written informed consent.

### Apparatus

Experiments were conducted at the *Spatial Audio Lab* (SAL) of the Center for Interdisciplinary Research in Music Media and Technology (*CIRMMT*). The lab is an anechoic room of 5.40 m (W) × 6.40 m (L) × 3.60 m (H). We measured reverberation time (0.09 s), early decay time (0.28 s) and background noise (23 dBA).

Stimuli were presented over a circular array of 24 loudspeakers regularly spaced on a circle (of radius 1.8 m) in the horizontal plane at ear level (see Fig. 3a). Participants sat on a chair with their head positioned in the center of the circular array of 24 Genelec 8030 A with a flat frequency response between 58 Hz and 20 kHz ± 2 dB. Stimuli were played on an Apple MacPro (Apple, Cupertino, CA) using an RME Madi HDSPe Sound card (Haimhausen, Germany) connected to an RME M32 DA digital-analog converter (Haimhausen, Germany). All devices were located outside the listening room to minimize background noise. The task was done with dimmed lights.

We used Vector Base Amplitude Panning (VBAP) spatialization for positioning virtual sound sources between speakers^36^. However, we compute the position for each audio sample, *i.e*. 44100 instead of 1000 samples per second in the original implementation^36^.

## Supplementary information

Supplementary Information for Auditory motion perception emerges from successive sound localizations integrated over time
