# Supplementary material for: Auditory motion perception emerges from successive sound localizations integrated over time
Source: Sci Rep. 2019 Nov 11;9:16437. doi: 10.1038/s41598-019-52742-0 (PMC6848124; doi:10.1038/s41598-019-52742-0)
Supplement: Supplementary file 1 — Supplementary Information for Auditory motion perception emerges from successive sound localizations integrated over time [file 41598_2019_52742_MOESM1_ESM.pdf]

Supplementary Information

Auditory motion perception emerges from successive sound localizations integrated over time

Vincent Roggerone[1,*], **Jonathan Vacher**[2], **Cynthia Tarlao**[1], **and Catherine Guastavino**[1]

[1]Centre for Interdisciplinary Research in Music Media and Technology, Multimodal Interaction Laboratory, McGill University, Montreal, Canada
[2]Department of Systems and Computational Biology, Albert Einstein College of Medicine, New-York, USA
[*]corresponding author : roggerone.vincent@live.fr

Effect of Sound Pressure Level on the Upper Limit

In addition to the effect of spectral content, we investigate the effect of sound pressure level on the Upper Limit by manipulating the level of the stimuli presented in Exps. 1 and 2 in additional conditions.

Experiment 1 : We present a White Noise (WN) at 2 different levels, namely (30 dB) and (60 dB), used as the reference stimulus. A T-test with Bonferroni correction indicates a significant difference between the two level conditions. However, the low level condition was only only 7 dB above the background noise and many participants reported that the stimulus was sometimes inaudible. We thus increased the level in Experiment 2.

Experiment 2 : We further investigate the effect of level by presenting WNs at 35 dB, (50 dB), and 60 dB. Results are summarized in figure 1. A repeated-measure ANOVA reveals a main effect of level ($F(2,30) = 5.179$, $p = 1.2 \times 10^{-3}$). Posthoc tests indicate that the UL at WN50dB was significantly higher than at WN35dB (p < 0.023, Mean difference : 0.234) but WN60dB was not significantly different from the other two.

We conclude that the significant differences observed in Exp. 1 with the WN at 30 dB and the WN at 60 dB is due to insufficient signal to noise ratio. In Experiment 2 we only observed significant difference between the WN at 35 dB and the WN at 50 dB, but not with WN at 60 dB . This suggests the observed differences are not reliable and do not reflect a true effect of the SPL, in line with the lack of effect of level reported on static localization[1].

Comparison with previous findings by Féron et al.[2]

Our data demonstrates the effect of spectral content on the UL, which increases with BW and CF. This result might seem in contradiction with those from[2], shown in Figure 3, who reported higher ULs for low-pitched sounds than for high-pitched sounds using harmonic sounds. But a closer look at the stimuli used explains this discrepancy. We present a time frequency analysis of the four harmonic sounds used (see Figure 3). The analysis reveals that high-pitched sounds had a narrower BW (as the sounds had no energy above 5 kHz, see Table 1). Based on our findings, we predict lower ULs for high-pitch sounds since their BW are narrower. Féron et al.[2] also used Band Limited Noises but failed to observe differences across noises, which might seem inconsistent with our findings. The difference can however be attributed to the filters used: while we used eighth-order filters with very steep slope, Féron used second-order filters. As a result, all their Band Pass Limited noise contained the high-frequency content necessary to achieve optimal UL, which explains their results.

Model implementation details

The psychometric relation between Q_x and the front-back confusion rate

We use the following psychometric function

$$S_r(Q_x, e, \alpha, \beta) = 1 - e + \frac{e - 0.5}{2}\left(1 + \operatorname{erf}(\alpha \log(Q_{\max} - Q_x) - \beta \log(Q_x))\right) \quad (1)$$

Such a function has the standard S-shape of a logistic function and is adapted to a bounded support $[0, Q_{\max}]$. Parameters α and β control the slope and the asymmetry of the graph while e controls the maximum achievable success rate. The parameters e, α and β are obtained by non-linear least squares regression on Langendijk's[3] data (`lsqcurvefit` function in matlab).

Frequency interpolation

The gradient along frequencies is computed using finite differences. In order to keep the number of central frequency samples equal to the number of gradient samples, we use the interpolated frequency samples $(0.5(f_n + f_{n+1}))_{n \in \{1, N-1\}}$.

Numerical issues in spectral gradient computations

The computation of the spectral gradient in Equation 4 of the main text suffers from numerical issues because differences in log-energy can be high when the energy is close to zero. Perceptually, these gradients are masked by the background noise. To address this problem, we apply the filtering of the sound x only after the gradient computation. To do so, we set to zero the gammatone bands with a central frequency outside the cut-off frequencies. In addition, for the two gammatone bands near the cut-off frequencies, we approximate the remaining energy by linear interpolation with the energy of the gammatone band immediately after the cut-off frequency.

For example, in the case of a band-pass filter with a high cut-off frequency f_h such that $f_i < f_h < f_{i+1}$ where f_i is the central frequency of the i-th gammatone. The approximate log-energy gradient is given by

$$\hat{P}_x(\theta, n) = \begin{cases} P_x(\theta, n) \text{ if } n \leq i \\ \frac{f_h - f_i}{f_{i+1} - f_i} P_x(\theta, n) \text{ if } n = i+1 \\ 0 \text{ if } n > i+1 \end{cases} . \quad (2)$$

Model discussion

Binaural *v.s.* monaural model of spectral cues

Our model is formally binaural because of Equation 3 in the main text. An alternative would be to consider both ears as independent. In this case, one would compute the front-back cues for both ears and sum them $Q_x = Q_x^r + Q_x^l$. As such the model would combine two monaural cues. However, this model would involve more numerical issues than those raised in section 3.C : considering a single ear, the HRTF energy in the controlateral directions is very low, potentially resulting in high values for the computed gradient. In addition, such a model would require a binaural weighting such as the one used by Majdak[4]. Our implementation solves both issues.

References

1. Yost, W. A. Sound source localization identification accuracy: Level and duration dependencies. The J. Acoust. Soc. Am. **140** (2016).
2. Féron, F.-X., Frissen, I., Boissinot, J. & Guastavino, C. Upper limits of auditory rotational motion perception. The J. Acoust. Soc. Am. **128**, 3703–3714 (2010).
3. Langendijk, E. H. A. & Bronkhorst, A. W. Contribution of spectral cues to human sound localization. The J. Acoust. Soc. Am. **112**, 1583–1596 (2002).
4. Majdak, P., Baumgartner, R. & Laback, B. Acoustic and non-acoustic factors in modeling listener-specific performance of sagittal-plane sound localization. Front Psychol. **5** (2014).
5. Camier, C., Boissinot, J. & Guastavino, C. On the robustness of upper limits for circular auditory motion perception. J. on Multimodal User Interfaces **10**, 285–298 (2016).

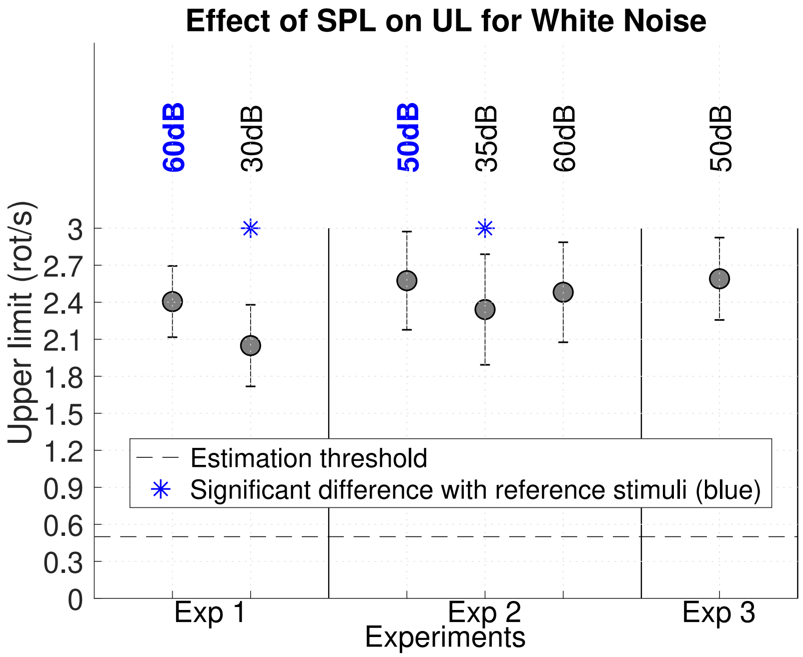

Figure 1. UL estimated with WN stimuli at different sound levels across all experiments.

	Label	Low cut-off frequency (kHz)	High cut-off frequency (kHz)	Sound Pressure Level (dBA)	Starting speed (rot/s)	Filter type
Exp 1	250Hz 2oct	0.1	0.6	61	0.5	Band Pass
	250Hz 4oct	0.06	1.06	65	0.5	
	2kHz 2oct	0.83	4.83	65	0.9	
	2kHz 4oct	0.47	8.47	63	0.9	
	4kHz 2oct	1.65	9.66	62	1.3	
	4kHz 4oct	0.94	16.94	58	1.3	
Exp 2	4kHz 1/2oct	3.1	5.1	55	0.5	Band Pass
	4kHz 1oct	2.5	6.5	51.5	0.5	
	4kHz 2oct	1.6	9.6	50.3	1.3	
	4kHz 3oct	1.2	13.2	49.5	1.3	
	4kHz 4oct	0.9	16.9	48.2	1.3	
Exp 3	<BS>4-16kHz	4	16	50.1	0.9	Band Stop
	<BS>4-8kHz	4	8	49.9	1.3	
	<BS>5.7-11.3kHz	5.7	11.3	50.4	1.3	
	<BS>8-16kHz	8	16	51.0	1.3	
	<BS>5.7-8kHz	5.7	8	49.8	1.3	
	<BS>8-11.3kHz	8	11.3	49.7	1.3	
	<BS>11.3-16kHz	11.3	16	50.5	1.3	
Féron	HT330	0.33	5	60-65	n/a	Harmonics
	HT440	0.44	5	60-65	n/a	
	HT880	0.88	5	60-65	n/a	
	HT1760	1.76	5	60-65	n/a	

Table 1. Table of parameters of the stimuli used and the measured level of presentation for the 3 experiments and the one used by Féron. Levels were adjusted to have the same perceptive level.

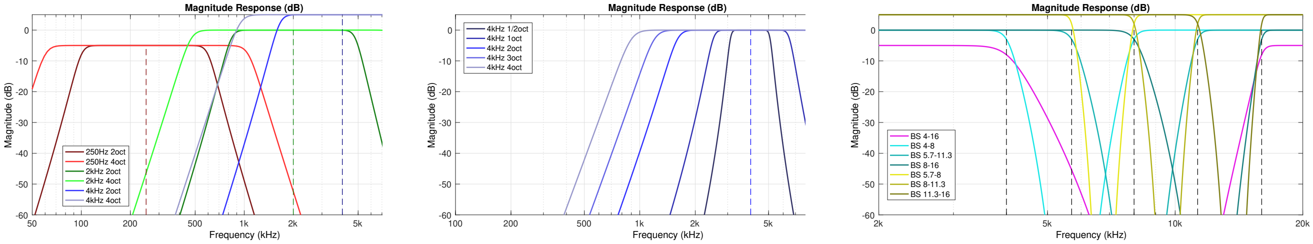

(a) Filters used to generate the stimuli in experiments 1, 2 and 3 (from left to right).

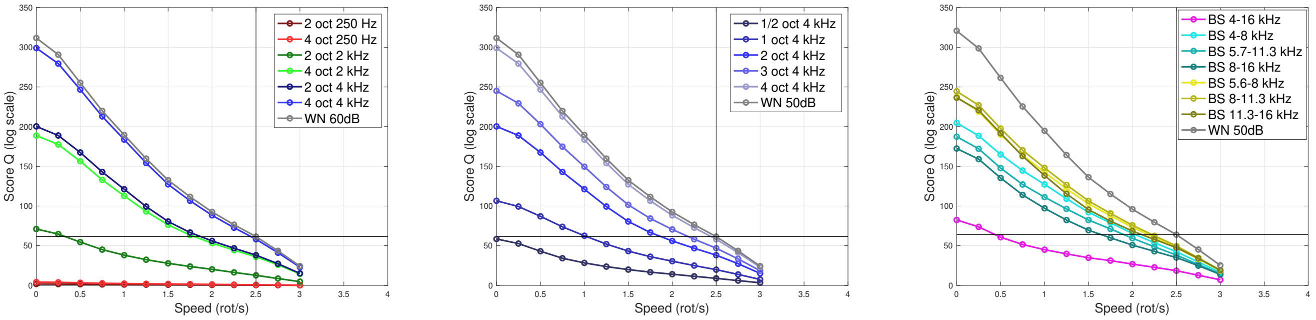

(b) Functions $\omega \mapsto Q_x(\omega)$ of the sound x in experiments 1, 2, and 3 (from left to right).

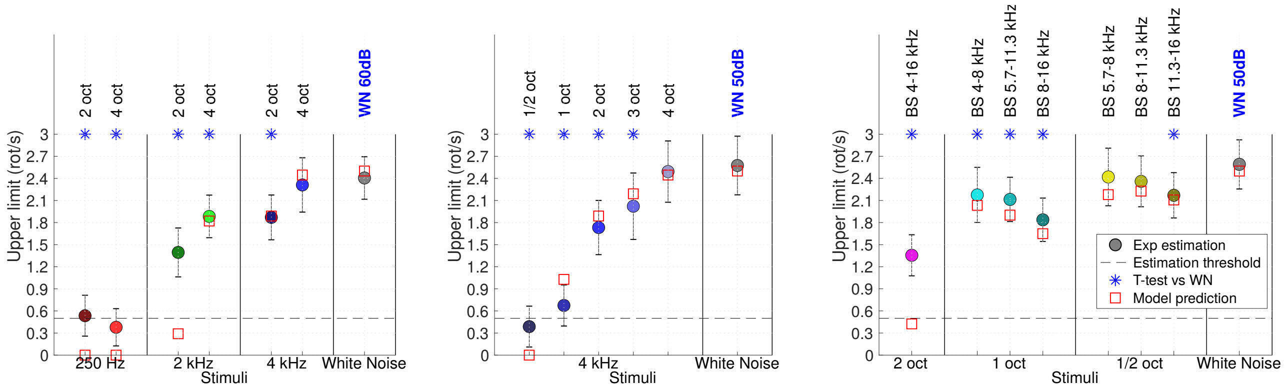

(c) Experimental results and model predictions (same figure as in the main text)

Figure 2. Filters used for the experiment, functions $\omega \mapsto Q_x(\omega)$ and UL results.

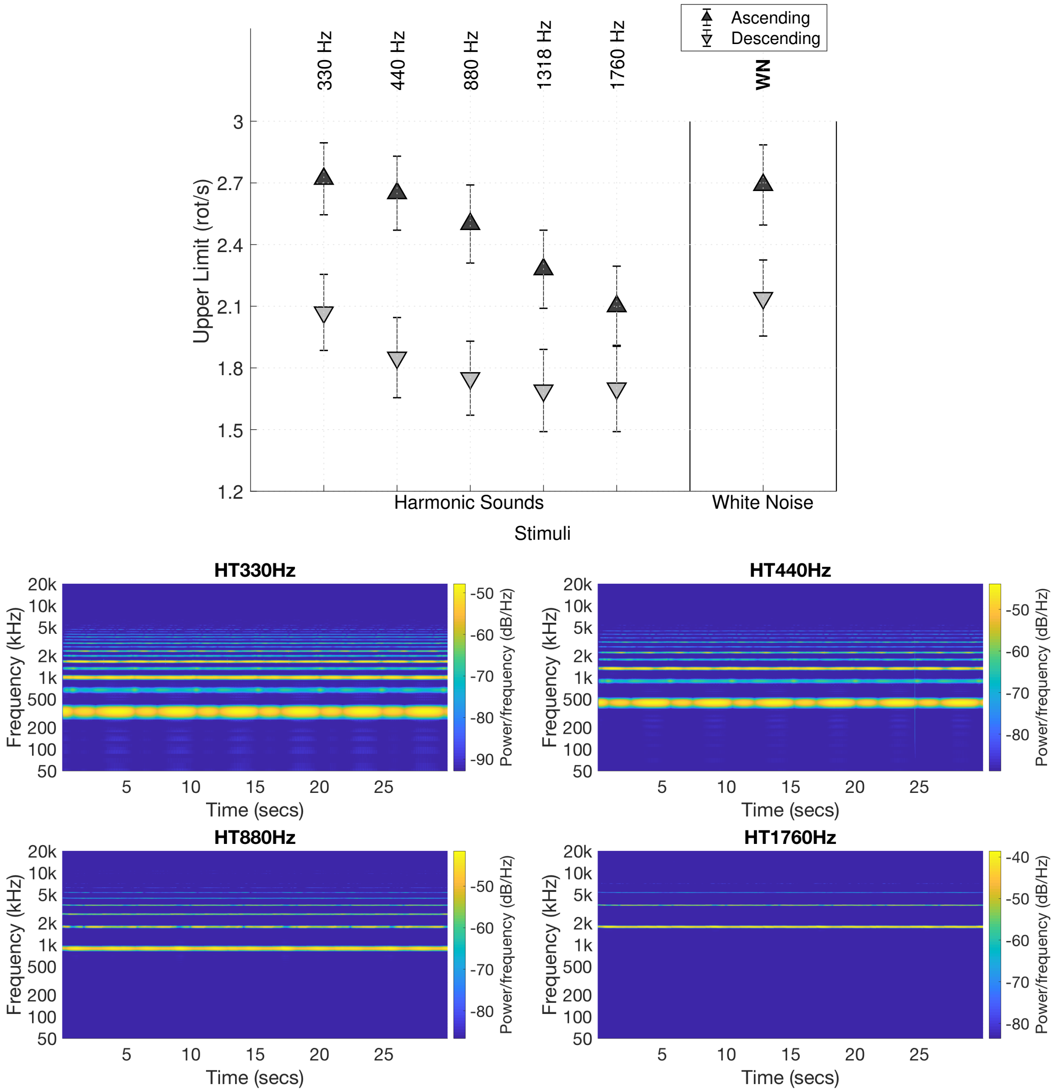

Figure 3. Top: UL obtained by Féron *et al.* adapted from Fig. 2[2]. The estimation method of the UL is different from ours but the UL has been shown to be robust to different estimation methods[5]. In Féron[2], the sound was either accelerating or decelerating, and participants were asked to indicate when they were unable (or respectively, able) to perceive the direction, hence the strong hysteresis observed. Bottom: Spectral content of Féron's harmonics sounds with fundamental frequencies: 330 Hz, 440 Hz, 880 Hz, and 1760 Hz. We use a -50 dB threshold for the time-frequency representation, which is below the absolute threshold of audition. Analyses show that the higher the pitch, the narrower the BW, explaining the observed decrease in UL.
